# Heterogeneity of work alienation and its relationship with job embeddedness among Chinese nurses: a cross-sectional study using latent profile analysis

**DOI:** 10.1186/s12912-023-01674-2

**Published:** 2024-02-15

**Authors:** Changchang Chen, Xutong Zheng, Yi Cui, Hezi Mu, Qian Yang, Man Zhang, Huan Xu, Jian Guan, Wenjie Chen, Hongjuan Lang

**Affiliations:** 1 Department of Nursing, Air Force Medical University, Xi’an, Shaanxi China; 2 School of Nursing, China Medical University, Shenyang, Liaoning China; 3 Intensive Care Unit, Shaanxi Provincial People’s Hospital, Xi’an, Shaanxi China; 4 School of Nursing, Shaanxi University of Chinese Medicine, Xian Yang, Shaanxi China; 5 Intensive Care Unit, Sixth Affiliated Hospital of Harbin Medical University, Harbin, Heilongjiang China; 6 College of Chemical and Biological Engineering, Yichun University, Yichun, Jiangxi China

**Keywords:** Work alienation, Job embeddedness, Latent profile analysis, Nurse, China

## Abstract

**Objective:**

To identify the distinct profiles of work alienation among Chinese nurses, examine the demographic factors associated with profile memberships, and then explore the relationship between latent categories of work alienation and job embeddedness.

**Methods:**

A cross-sectional survey of 523 nurses was conducted from July to August 2023. Latent profile analysis (LPA) was performed to identify distinct profiles of nurses based on three aspects: powerlessness, helplessness, and meaningfulness. A multinomial logistic regression analysis was conducted to explore the predictors of profile membership. Hierarchical regression analysis was carried out to examine the association between profile memberships and job embeddedness.

**Results:**

Three subgroups of work alienation of nurses were identified: 23.1%, 57.8%, and 19.1% in the low work alienation group (profile 1), the moderate work alienation group (profile 3), and the high work alienation group (profile 2), respectively. Nurses with college degrees were more likely to be grouped into moderate work alienation. Nurses who did not work night shifts were more likely to have low or moderate levels of work alienation. Nurses earning 2,000–3,000 and 3,001–5,000 yuan per month were likely to be in the low work alienation group. The different categories of work alienation significantly predicted job embeddedness among nurses (Δ*R*^2^ = 0.103, *p* < 0.001).

**Conclusions:**

Work alienation has an important impact on clinical nurses’ job embeddedness. Nursing managers should pay attention to the differences in individual work alienation status and adopt reasonable management strategies to improve the level of job embeddedness, ensure the quality of care, and reduce nursing turnover.

**Supplementary Information:**

The online version contains supplementary material available at 10.1186/s12912-023-01674-2.

## Background

Around the globe, nursing is an essential part of health care, and nurses play a key role in achieving universal health coverage (UHC) and the sustainable development goals (SDGs) as well as improving the population health outcomes [1]. However, the number and distribution of nurses worldwide is not compatible with UHC and SDGs. The shortage of nurses was estimated to be 5.9 million in 2018 and to reach 5.7 million by 2030 [2]. In China, the nursing shortage is also a critical issue. By the end of 2021, there were 3.56 nurses per 1,000 people in mainland China, far lower than that in high-income countries [2, 3]. Despite the high demand for nurses, there is still a considerable proportion of nursing staff with turnover intention. A multi-center cross-sectional study in China found most nurses (69.4%) had a high level of turnover intention [4]. Notably, nurses’ turnover intention poses a severe threat to the quality of care and the development of nursing careers. Furthermore, the increasing proportion of turnover increases the workload of other nurses, which may lead to lower job satisfaction and embeddedness for nurses in the position [5]. Therefore, to early predict nurses’ turnover intention and reduce the loss of nurses, it is necessary to identify job embeddedness.

Miriam et al. have presented job embeddedness as a broad constellation of influence on employee retention [6]. Unlike the traditional turnover model, job embeddedness focuses on the reasons why nurses want to stay in their current jobs rather than the causes of turnover, which is a new approach to elucidating the causes of retention and a key construct in mediating employee retention [7]. It is defined as a net or a web in which employees are trapped, representing the degree of deep connections and rootedness of an individual with their job [8]. There are three critical aspects of job embeddedness: (a) links refer to the extent to which nurses are connected to their colleagues or organizational activities. The higher the number of links between an individual and his/her network, the more connected he/she is to the work and the organization; (b) fit refers to the perceived organizational compatibility or comfort of nurses in their clinical work. Nurses’ values, professional goals, and plans must fit with the prevailing culture and work requirements of the hospital; (c) sacrifice is the material and psychological losses perceived by clinical nurses as a result of leaving a job, including work of interest, coworkers they get along with, unit salary, and benefits [6, 9]. Globally, healthcare organizations are striving to retain and embed nurses in their workplaces. Nurses with high job embeddedness are more likely to exhibit high levels of affective commitment, organizational citizenship behavior, job performance, innovation, and job satisfaction [10–12]. In addition, job embeddedness increases nurses’ attachment and retention to the hospital, thereby alleviating nurse shortages and turnover. Therefore, attention should be paid to exploring job embeddedness and other psychological influences to help hospital administrators take active action to strengthen job embeddedness.

Previous studies have shown that work alienation is a precursor to decreased work motivation propensity and is a widespread phenomenon in work life among nurses [5]. It refers to the state of psychological separation in which the work situation is perceived to lack the potential for meeting the needs or expectations of the employee [13]. As an important indicator of the negative psychological state, work alienation mainly includes powerlessness, helplessness, and meaninglessness. Powerlessness is a feeling that an individual has no control over the decision-making process. Individuals lack job autonomy and have limited freedom to control work activities [14–16]. Helplessness means the lonely feeling of a person who feels unable to fit in and be recognized and cared for by the organization they are in [17]. Meaninglessness is when an individual perceives his or her work as unimportant or worthless, or even fails to understand the relationship between his or her contribution and the organization’s goals [15, 16]. Nurses are prone to feelings of alienation due to heavy workloads, role uncertainty, inappropriate leadership styles, and promotion pressures. Previous studies have shown that when nurses’ work alienation is at a high level, it is related to decreased job satisfaction, low motivation, disharmonizing the nurse-patient relationship, and increased turnover intentions [18, 19].

**Fig. 1:**
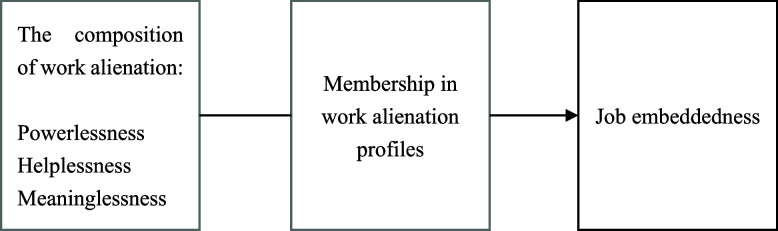
The relationship between work alienation profiles and job embeddedness

Social Exchange Theory (SET) serves as the theoretical foundation for our study, which is based on a potential reciprocity norm [20, 21]. According on SET, the relationship between nurses and organizations is also a social exchange relationship in nature. When the nurse’s expectations are met, a social exchange relationship is realized. The closer the interpersonal relationship, the stronger the employees’ sense of belonging to their work, thus creating greater value for the organization and achieving a win-win situation. However, when nurses are socio-emotionally and psychologically unfulfilled, it means that the exchange relationship between the two is alienated, thus reducing work commitment and the level of job embeddedness. Therefore, based on SET and literature analysis, we constructed a conceptual framework for this study (Fig. 1).

Previous research on nurses’ work alienation has mainly focused on the investigation of its current situation and related variables [22, 23]. There is still room for further investigation into whether there is heterogeneity within the work alienation group of nurses. There are no domestic or international studies that specifically address the relationship between work alienation and job embeddedness in nurses, and the deeper specific correlation mechanism between the two is not clear. In addition, most of the existing studies have used standardized scale scores or scale score thresholds to determine the level of work alienation in clinical nurses. However, this “variable-centered” approach neglects the variations brought about by individual differences, and there is a high degree of heterogeneity between subgroups assessed by this approach, that is, individuals with the same work alienation score responded in different patterns on each item. Therefore, to some extent, the findings of these studies do not truly represent the degree of work alienation among nurses.

Latent profile analysis (LPA), a person-centered approach, serves the following advantages in its usage. First, it allows us to identify the heterogeneity of the study population by categorizing potential subgroups [24, 25]. Nurses in each work alienation subgroup were homogeneous, whereas nurses in different work alienation subgroups were heterogeneous. It offers a better understanding of the population of nurses with different work alienation characteristics. Second, it reduces the requirement for otherwise complex higher-order interactions between variables in variable-centered approaches (requiring larger sample sizes to detect effects, uncertainty about the nature of how variables are combined within individuals, etc.) to a concise formulation [26–28]. Third, LPA has been used in many different psychological trait studies, e.g., exploring the meaning of life profile among nursing students [29]. It is possible to develop tailored-made interventions for sub-population to meet their needs better. Thus, by applying LPA, it is possible to identify previously unobserved subpopulations and thus better understand their hidden characteristics.

### Aims and hypotheses

This study aimed to identify the different work alienation profiles of nurses using LPA, examine the demographic factors associated with different alienation profiles, and then explore the relationship between latent categories and job embeddedness. The study is structured on the following three hypotheses:

- *Hypothesis 1*. There are subgroup differences in work alienation among nurses.
- *Hypothesis 2*. Nurses’ work alienation profiles vary across demographic and work-related characteristics.
- *Hypothesis 3*. Membership in different work alienation profiles can predict nurses’ job embeddedness.

## Methods

### Design

A cross-sectional design was conducted from July to August 2023. This study followed the STROBE (strengthening the reporting of observational studies in epidemiology) statement (see Appendix S1).

### Participants

A convenient sampling method was used to select nurses from 3 tertiary general hospitals in Xi’an, Yulin, and Xianyang, Shaanxi Province, China. The inclusion criteria were as follows: (a) nurses had completed the nurse registration and obtained the National Nursing Licensure Examination (NNLE); (b) participants could independently perform nursing duties; and (c) they were willing to participate in this survey. Head nurses, nursing interns, and nurses who were absent from their positions due to illness or vacation during the survey period were excluded.

### Sample size

Based on Kendall’s criteria of sample estimation, the sample size was calculated as 10 times the number of scale items [30]. In this study, a total of 31 items were included, which were the General Information Questionnaire (12 items), Nurses’ Work Alienation Questionnaire (12 items), and Global Job Embeddedness Scale (7 items). Therefore, the required minimal sample size was *N* = 10 * (12 + 12 + 7) = 310. Taking into account 10% to 20% of invalid questionnaires, the final simple size required was *N* = 310 ÷ (1–20%) ≈ 388. Combined with requirement of the sample size of at least 500 samples for LPA, we ultimately collected 561 questionnaires. Previous studies suggest that a sample size of 500 or larger is considered sufficient for LPA [31, 32]. Of the 561 questionnaires, a total of 523 responses were valid, resulting in an effective response rate of 93.23%.

### Measurement

#### Demographic questionnaire

The general demographic characteristics data were collected using a questionnaire specifically designed by the researchers. The data included gender, age, education, title, years of nursing work, department, days of night shift monthly, and salary.

#### Nurses’ Work Alienation Questionnaire

The Nurses’ Work Alienation Questionnaire was used to evaluate work alienation. The questionnaire was developed by Chinese scholar Ren Xiaojing, consisting of three dimensions and 12 items: powerlessness (4 items), helplessness (4 items), and meaninglessness (4 items) [33]. Each item is scored using a 5-point Likert scale ranging from 1 (not conforming) to 5 (very conforming). The total score ranges from 12 to 60, with higher scores indicating higher levels of alienation. The scale has been tested in Chinese nurses revealing acceptable reliability [34]. In this study, the Cronbach’s α coefficient of the total questionnaire was 0.939, and the Cronbach’s α coefficient of the three dimensions was 0.878, 0.907, and 0.921, respectively.

#### Global Job Embeddedness Item scale

The Global Job Embeddedness Items (GJEI) scale was developed by Crossley et al. [35] and translated by Zhang et al. [36]. It is widely used to assess the level of job embeddedness among healthcare professionals. The scale consists of a single dimension with a total of 7 items. All items are rated on a 5-point Likert scale ranging from 1 (strongly disagree) to 5 (strongly agree). Items 4 and 6 are reverse coded. The total score ranges from 7 to 35, with higher scores indicating greater job embeddedness among nursing personnel. In previous literature, a confirmatory factor analysis was performed on the scale showing good data fit (χ^2^ = 55.476, df = 12, χ^2^/df = 4.623, CFI = 0.990, TLI = 0.982, RMSEA = 0.060) [7]. The Scale has been validated in the Chinese populations [37]. Cronbach’s α were 0.850 and 0.802 in Zhou’s and Hu’s studies, respectively [38, 39]; in this study, Cronbach’s α for the nursing environment was 0.725.

### Data collection

The data were collected via an anonymous and self-reported questionnaire. Prior to the informational survey, a pre-survey was done with registered nurses in Xi’an (*n* = 10) to assess whether the questions could be easily understood and whether any technical problems existed using an online self-reported questionnaire (these data were not included in this study). The team leader was responsible for contacting the director of the hospital’s nursing department and explaining the purpose of the survey and the method of completing the questionnaire. With the consent and help of the hospital administrators, a link to an anonymous electronic questionnaire was sent to the hospital administrators through WeChat, which was then distributed to the clinical nurses. Consent and clear instructions were provided at the beginning of the questionnaires. During the questionnaire completion process, if any participant missed filling out certain sections, they would receive a prompt to complete the missing parts. This ensured the integrity of the questionnaire. We have set up IP addresses to limit responses from mobile phones and computers to only once, preventing duplicate submissions. Two uniformly trained investigators continuously monitored the recovery data. When no new data was generated for one consecutive week, the data was exported. Then two researchers double-checked the quality of the questionnaire. Any questionnaire that violated the requirements for completion (e.g., same answers for all items, unrealistic) was deleted. No materiality-based incentive was offered in this study.

### Data analysis

The data was analyzed using SPSS 26.0 and Mplus 8.3 software, and a bilateral test with a *p*-value < 0.05 was considered statistically significant. Descriptive statistics were computed for all demographic characteristics, including mean, standard deviation (SD), median, interquartile range (IQR), frequency, and percentage. The scores of the three dimensions of work alienation were standardized by SPSS 26.0 software to be used as external variables. Mplus 8.3 software was used to analyze the latent profile, which categorized the types of work alienation among nursing staff. The number of latent profiles was increased ranging from 1 to 5, and the best-fitting model was selected based on statistical and practical considerations [40, 41]. The following parameters were considered as criteria to determine the optimum number of classes: the Akaike Information Criterion (AIC), the Bayesian Information Criterion (BIC), and the sample-size adjusted Bayesian Information Criterion (aBIC) [42]. A smaller value indicates a better model fit [43]. The entropy value represents the accuracy of classification ranging between 0 and 1. An entropy value of 0.8 indicates a classification accuracy exceeding 90%, and the closer it is to 1, the higher the classification accuracy [44]. The likelihood ratio test indices (Lo-Mendell-Rubin, LMR) and the Bootstrap Likelihood Ratio Test (BLRT) based on Bootstrap were used to assess the fit differences of the latent profile models. If the *p*-value reaches the significance level, it indicates that a model with k categories significantly outperforms a model with k-1 categories [45].

After the best LPA solution was selected, participants were assigned to their most likely work alienation profile. Then, a multinomial logistic regression analysis was performed to explore the potential predictors previously identified in the literature as risk factors of work alienation among nurses. Odds ratio (OR) and 95% confidence interval (CI) were reported as effect estimates. The profiles of work alienation were as the dependent variable and the candidate predictor variables (demographic and work-related factors) were used as independent variables for multivariate logistic regression analysis.

Hierarchical regression analysis was employed to investigate the relationship between different patterns of work alienation and nursing staff’s job embeddedness while incorporating sociodemographic characteristics as covariates into the model. All variables with univariate *p*-values < 0.05 were chosen as independent variables for the multinomial regression models.

### Common method deviation test

Due to the self-report data collected in this study, there may be potential common method variance (CMV) [46]. In this study, we used several methods to prevent CMV, including the reverse item method, an anonymous questionnaire method, and randomization of item assignment across different constructs.

To examine the presence of common method bias (CMB), controlling for the effects of an unmeasured latent methods factor (ULMC) was used in this study. Based on the original model, a bifactor model was tested with the inclusion of a common method factor. The results of confirmatory factor analysis indicated minimal changes (< 0.05) in various fit indices after introducing the common method factor into the original model (ΔRMSEA = -0.015, ΔSRMR = -0.011, ΔCFI = 0.022, ΔTLI = 0.021). We found that the model did not significantly refine the fitting effect by controlling the common method factors, indicating that there is no serious CMB.

### Ethical considerations

This study was conducted following the ethical principles required in the Declaration of Helsinki and approved by the ethical committee of the Second Affiliated Hospital of Air Force Military Medical University. All participants were informed of the research objective and volunteered to participate in this survey, as well as having the right to refuse participation at any time.

### Result

#### Study population

The participants’ median age was 25 years (IQR 26–33). Most participants were female, had a bachelor’s degree or higher, held the title of senior nurse, and had been practicing nursing for between 1 and 5 years. Most worked in internal medicine, frequently worked night shifts (≥ 8 days per month), and earned 5001–7000 CYN per month. Table 1 summarizes the categorical characteristics of the 523 participants.

**Table 1:** Descriptive characteristics of the participants (*n* = 523)

Name of variable	Categories	N (%)
Gender	Male	17 (3.3)
	Female	506 (96.7)
Age (years) (R: 22–55)	20–30	301 (57.6)
	31–40	189 (36.1)
	> 40	33 (6.3)
Education	College degree	169 (32.2)
	Bachelor’s degree or above	354 (67.7)
Title	Primary nurse	153 (29.3)
	Senior nurse	246 (47.0)
	Nurse-in-charge and above	124 (23.7)
Years of nursing work	1–5	215 (41.1)
	6–10	177 (33.8)
	11–15	91 (17.4)
	> 15	40 (7.6)
Department	Internal medicine	299 (57.2)
	Surgery	88 (16.8)
	ICU/operating room/emergency department	73 (14.0)
	Others	63 (12.0)
Days of night shift monthly	0	58 (11.1)
	1–7	160 (30.6)
	≥ 8	305 (58.3)
Monthly income (CNY)	2000–3000	100 (19.1)
	3001–5000	173 (33.1)
	5001–7000	180 (34.4)
	> 7000	70 (13.4)

*N (%)* Number and percentage, *R* Range, *ICU* Intensive care unit, *CNY* Chinese Yuan

### Latent profile analysis of work alienation in Chinese nurses

#### Results of latent profile analysis (LPA)

In this study, a total of five latent profile models were explored, and their fitting indexes of different profiles were presented in Table 2. As the number of latent profiles increased, the values of AIC, BIC, and aBIC gradually decreased. However, the LMRT was not significant when the profile was divided into four categories, indicating that the three-profile model outperformed the four-profile model. Considering that each category should account for at least 5.0% of the total sample size, the five-profile model had a profile with a very small proportion, making it impractical to adopt. Upon comparison, the three-profile model demonstrated lower AIC, BIC, and BIC values than the two-profile model, with a higher entropy value of ≥ 0.8. The average attribution probabilities of the three categories in Model 3 were 90.1%, 96.9%, and 91.6% respectively (see Table 3), and the LMR and BLRT for the category model were statistically significant, indicating the best fit for Model 3. Considering the comprehensive analysis mentioned above, selecting a three-class solution yielded the optimal classification result for work alienation among nursing staff.

**Table 2:** Latent profile analysis of work alienation with model fit results (*n* = 523)

Profile	k	LL	AIC	BIC	aBIC	Entropy	LMRT (*P*)	BLRT (*P*)	Proportion
1	6	-4375.185	8762.369	8787.927	8768.881	1.000	-	-	1
2	10	-4153.638	8327.276	8369.872	8338.129	0.804	< 0.001	< 0.001	71.0%/29.0%
**3**	**14**	**-4030.770**	**8089.541**	**8149.175**	**8104.736**	**0.832**	**< 0.001**	**< 0.001**	**23.1%/19.1%/57.8%**
4	18	-4001.698	8039.396	8116.068	8058.932	0.847	0.107	< 0.001	20.0%/21.6%/5.0%/53.7%
5	22	-3949.741	7943.482	8037.192	7967.359	0.935	0.002	< 0.001	14.1%/37.7%/25.6%/4.8%/17.8%

*k* The Free parameters, *LL* The Log likelihood, *AIC* Akaike information criterion, *BIC* Bayesian information criterion, *aBIC* Sample-size adjusted Bayesian information criterion, *LMRT* Lo-Mendell-Rub test, *BLRT* Bootstrap likelihood ratio test

**Table 3:** Average probability of attribution for each potential profile

Class	Profile 1(%)	Profile 2 (%)	Profile 3 (%)
Profile 1	0.901	0.000	0.099
Profile 2	0.000	0.969	0.031
Profile 3	0.051	0.033	0.916

#### Naming of three potential work alienation types

**Fig. 2:**
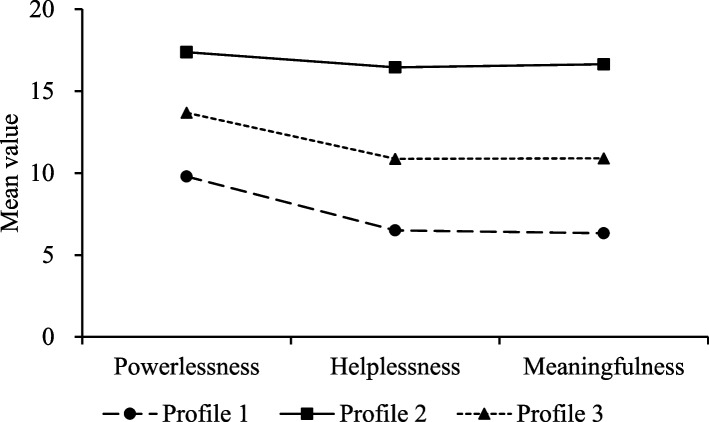
Mean value characteristics for the three-profile solution

Figure 2 shows the scores on three work alienation dimensions for each profile. The x-axis shows the baseline work alienation characteristics and y-axis presents the mean score. As shown in Fig. 2, nurses in Profile 1 (‘low alienation’) had the lowest scores on all three dimensions of work alienation, accounted for 23.1% (*n* = 121) of all participants, while those in Profile 2 (‘high alienation’) had the highest scores, accounting for approximately 19.1% (*n* = 100) of the total sample. Profile 3 (‘moderate alienation’) had scores significantly higher than Profile 1 but lower than Profile 2, comprising approximately 57.8% (*n* = 302) of the total participants.

#### Predictors of latent profiles of work alienation

Next, we explored whether nurses divided into specific profiles differed in terms of potential indicators using multinomial logistic regression analysis (see Table 4). The profiles of work alienation were as the dependent variable and the candidate predictor variables (demographic and work-related factors) were used as independent variables. The high work alienation group (second profile) was as the reference. There were no significant interactions between variables, there were no outliers. The results showed a significant effect of the days of night shift monthly and monthly income on the low work alienation group (first profile). There was a statistically significant effect of education and days of night shift monthly on the moderate work alienation group (third profile). No meaningful differences emerged between the heterogeneous subgroups in terms of other examined variables.

**Table 4:** Results of multivariate logistic regression analysis (*n* = 523)

Variables	Low work alienation VS. High work alienation					Moderate work alienation VS. High work alienation				
	*β*	SE	OR	95% CI	*P*	*β*	SE	OR	95% CI	*P*
**Gender (ref: female)**										
Male	-0.307	0.774	0.735	0.161 ± 3.350	0.691	-0.655	0.643	0.520	0.147 ± 1.832	0.308
**Age (ref: > 40)**										
20–30	-1.229	1.020	0.293	0.040 ± 2.163	0.229	-0.532	0.930	0.587	0.095 ± 3.636	0.567
31–40	-1.509	0.949	0.221	0.034 ± 1.419	0.112	-0.470	0.881	0.625	0.111 ± 3.516	0.594
**Education (ref: bachelor’s degree or above)**										
College degree	-0.363	0.328	0.696	0.366 ± 1.322	0.268	-0.637	0.276	0.529	0.308 ± 0.907	**0.021**
**Title (ref: nurse in charge and above)**										
Primary nurse	-0.163	0.701	0.850	0.215 ± 3.360	0.817	-0.324	0.562	0.723	0.240 ± 2.174	0.564
Senior nurse	0.076	0.522	1.079	0.388 ± 3.001	0.885	0.091	0.412	1.095	0.489 ± 2.454	0.826
**Years of nursing work (ref: > 15)**										
1–5	-1.179	1.023	0.308	0.041 ± 2.283	0.249	-0.076	0.881	0.926	0.165 ± 5.210	0.931
6–10	-0.413	0.928	0.662	0.107 ± 4.078	0.656	-0.161	0.810	0.852	0.174 ± 4.162	0.843
11–15	0.145	0.907	1.157	0.196 ± 6.837	0.873	0.058	0.818	1.059	0.213 ± 5.263	0.944
**Department (ref: others)**										
Internal medicine	0.425	0.487	1.529	0.589 ± 3.971	0.383	0.220	0.403	1.246	0.565 ± 2.745	0.585
Surgery	0.155	0.576	1.168	0.378 ± 3.611	0.788	0.218	0.469	1.244	0.497 ± 3.117	0.641
ICU/operating room/emergency department	1.084	0.621	2.957	0.875 ± 9.995	0.081	0.839	0.532	2.313	0.815 ± 6.565	0.115
**Days of night shift monthly (ref: ≥ 8)**										
0	1.214	0.609	3.365	1.020 ± 11.100	**0.046**	1.426	0.515	4.162	1.516 ± 11.422	**0.006**
1–7	0.481	0.351	1.617	0.813 ± 3.216	0.170	0.489	0.287	1.631	0.929 ± 2.864	0.089
**Monthly income (CNY) (ref: > 7000)**										
2000–3000	1.902	0.605	6.702	2.048 ± 21.932	**0.002**	0.167	0.538	1.182	0.412 ± 3.391	0.756
3001–5000	-1.219	0.546	0.296	0.101 ± 0.863	**0.026**	-0.816	0.424	0.442	0.192 ± 1.016	0.054
5001–7000	0.487	0.545	1.628	0.559 ± 4.738	0.371	-0.441	0.455	0.643	0.264 ± 1.570	0.333

*Ref* Reference, *SE* Standard error, *OR* Odds ratio, *CI* Confidence interval, *ICU* Intensive care unit, *CNY* Chinese Yuan

#### Association between latent-profile membership of work alienation and job embeddedness of nurses

There were also significant differences in the latent profiles of nurses’ work alienation concerning their mean scores in job embeddedness (*p* < 0.001) (see Table 5). The mean scores of the job embeddedness of nurses in profiles 1, 2, and 3 were 26.87 (SD = 4.141), 23.75 (SD = 3.239), and 23.40 (SD = 3.853), respectively. Moreover, the SNK test revealed that the mean score of the ‘low work alienation’ group was significantly lower than that of the ‘moderate work alienation’ group and the ‘high work alienation’ group, whereas there is no significant difference between profile 2 and profile 3.

A hierarchical regression analysis was conducted to explore the impact on nurses’ job embeddedness. Job embeddedness was treated as the dependent variable, with a two-layer approach. In the first layer, control variables such as age, gender, and department were included. In the second layer, the latent categories of work alienation were introduced. The first item of the independent variables was set to the reference group. The results revealed significant effects of different latent categories of work alienation on job embeddedness. The results of hierarchical regression analysis indicated that age and monthly income entered the regression equation model for job embeddedness (*F* = 4.727, *R*^*2*^ = 0.044, *p* < 0.001). Based on Model 1, distinct subgroups (profile 1 vs. profile 2: *β*=-0.278, *p* < 0.001; profile 1 vs. profile 3: *β* = -0.409, *p* < 0.001) were significantly associated with job embeddedness. Compared with those in profile 1, nurses in profiles 2 and 3 had a significantly lower job embeddedness score. The overall model was significant (*F* = 12.690, *p* < 0.001), and the subgroups of work alienation explained an additional 10.3% of the variance in the job embeddedness (Δ*R*^*2*^ = 0.103), with the overall model explaining 13.6%. The result of hierarchical linear regression analysis for the job embeddedness is shown in Table 6.

**Table 5:** Job embeddedness difference of three profiles

	Job embeddedness (M ± SD)
Low work alienation (profile 1)	26.87 ± 4.141
Moderate work alienation (profile 3)	23.40 ± 3.853
High work alienation (profile 2)	23.75 ± 3.239
*Hc*	56.560
*P*	< 0.001
*SNK*	1 > 2, 1 > 3

*M* Mean, *SD* Standard deviation, *SNK* Student-Newman-Keuls

**Table 6:** Hierarchical linear regression analysis for the job embeddedness (*n* = 523)

Variables	Model 1					Medel 2				
	*B*	*SE*	*β*	*t*	*P*	*B*	*SE*	*β*	*t*	*P*
Contant	24.839	0.429		57.878	0.000	26.696	0.470		56.755	0.000
Age										
31–40	1.026	0.408	0.121	2.512	0.012	0.886	0.388	0.105	2.285	0.023
> 40	2.436	0.745	0.146	3.270	0.001	1.652	0.713	0.099	2.315	0.021
Monthly income										
3001–5000	-1.472	0.508	-0.170	-2.901	0.004	-0.289	0.506	-0.033	-0.570	0.569
5001–7000	-1.262	0.507	-0.148	-2.487	0.013	-0.646	0.487	-0.075	-1.325	0.186
> 7000	-1.290	0.635	-0.108	-2.032	0.043	-0.295	0.614	-0.025	-0.481	0.631
Work alienation										
Moderate work alienation (profile 3)						-3.367	0.428	-0.409	-7.865	0.000
High work alienation (profile 2)						-2.874	0.537	-0.278	-5.357	0.000
*F*			4.727**					12.690**		
*R*^*2*^			0.044					0.147		
Adjusted *R*^*2*^			0.034					0.136		
Δ*R*^*2*^								0.103**		

**p < 0.001; *SE* Standard error; Δ*R*^*2*^, Change in *R*^2^

## Discussion

This study identified the heterogeneity of work alienation among Chinese nurses, examined the demographic factors associated with profile memberships, and then explored the relationship between latent categories of work alienation and job embeddedness. To our knowledge, our study is the first to utilize a person-centered approach to examine the alienation of nurses in clinical work and the potential outcomes of different alienation levels.

### Potential profile characteristics of work alienation among Chinese nurses

According to the model predictions of the LPA, three distinct work alienation profiles were depicted in our results that best fit our model. Nurses in the high work alienation group have a high level of powerlessness, helplessness, and meaningfulness. This may be due to problems such as the nursing shortage, coupled with the increasing demand for quality of care and the competitive pressure for promotion in recent years. The Conservation of Resources (COR) theory argues that losses have a stronger impact than gains [47]. In line with COR theory, the internal and external organizational stressors (e.g., increased workload, decreased autonomy) faced by nursing staff in their clinical work can be seen as a threat to these resources (i.e., the nurses’ energetic, physical, emotional, and psychological resources) because it puts these resources at risk of exhaustion. Resource exhaustion can arouse a sense of alienation among employees from their work, leading them to reduce their interactions in the workplace to prevent further exhaustion of resources and to protect their remaining resources. This category of population is the most noteworthy one. Nursing managers should create fair and supportive work environments, and develop a standardized process for making decisions on major matters involving nurses’ rights and interests, such as fair compensation, growth opportunities, support, and rewards, to reduce nurses’ sense of alienation and stabilize the nursing team.

The low and moderate level groups show approximately the same trend as can be seen in Fig. 2. The sense of powerlessness dimension had a higher mean score than the other dimensions in general, which is consistent with previous research findings [18]. This may be because nurses feel responsible for alleviating the suffering of patients. However, due to the limitations of various reasons such as medical conditions and the nature of the disease, they are powerless to do more for their patients and are frustrated by their inability to proactively seek solutions to their problems. Compared with the powerlessness dimension, the mean score of the helplessness dimension is lower, which may be related to the enhancement of the humanistic care management concept, so that the overall working environment of nurses can be improved, and nurses can get more help and care from colleagues and managers. The meaninglessness dimension also scored relatively low, indicating that this group of nurses perceived their work as having a sense of value and a high level of professional identity. This is probably because this group has a higher subjective social status, perceives the gains and benefits of the profession, and agrees that practicing nursing can promote their growth and development [48]. In this subgroup of nurses, although the nurses have a relatively low level of alienation, they still need attention and timely nursing intervention to prevent further progression.

### Factors influencing latent categories of work alienation among nurses

The second aim of this study was to investigate whether these profiles have meaningful demographic and work-related indicators, which might help managers identify different levels of work alienation early and provide timely interventions to reduce nurse turnover and improve the quality of care. The demographic and work-related predictors of profile membership include education, the times of night shift, and the income of nurses.

Nurses with college degrees were more likely to be grouped into moderate work alienation rather than high work alienation groups. This result is consistent with earlier research findings that higher levels of education were correlated with higher levels of work alienation [5]. Nurses with higher education levels are usually engaged in a wide range of jobs such as clinical care, scientific research, teaching, and management. These overloaded tasks occupy excessive personal rest time, which reduces their professional identity and job satisfaction [5]. Compared to highly educated nurses, nurses with low education have lower self-orientation, and lower expectations and demands for compensation, professional development, and empowerment. In this case, the conditions provided by the clinic are more likely to match the nurses’ expectations and needs, and therefore the sense of alienation is lower. However, the impact of education on nurse burnout, job dissatisfaction, and turnover intentions among registered nurses have been controversial [49], which warrants continuing exploration in the future.

Nurses who did not work night shifts were more likely to have low or moderate levels of work alienation, consistent with previous research. This may be because nurses who do not work night shifts have more regular circadian rhythms and sleep-wake cycles, higher quality sleep, longer sleep hours, and more time with parents and children, thus reducing negative emotions. There is mounting evidence that the disruption to circadian rhythm in night shift nurses impacts job performance [50, 51]. Instead, nurses without night shifts reported fewer patient care errors, fewer workplace injuries, and a lower risk of sprains or strains [52, 53], and thus lower negative psychology and sense of alienation.

In addition, nurses earning 2,000–3,000 and 3,001–5,000 yuan per month were likely to be in the low work alienation group. This is inconsistent with the findings of You et al., which showed that nurses’ work alienation scores decreased progressively with increasing income because a high salary represents, to some extent, the ability of nurses to realize their professional values, which leads to high job satisfaction and professional identity [5]. In this study, nurses with low salaries are more likely to have low levels of alienation, which may be related to the region. The survey showed that Xi’an, Yulin and Xianyang were selected as “2023, 2022 and 2021 China’s Happiest Cities Ranking” respectively, ranking among the top prefecture-level cities in China. These regions are rich in education, medical care, culture, and transportation resources, and have certain advantages in terms of quality of life, ecological environment, and sustainable development, resulting in a high happiness index for residents. Furthermore, with COVID-19 no longer a public health emergency of international concern [54], multiple stressors for nurses, including increased workload demands, high risk of infection, separation from families are greatly alleviated.

### Latent profile differences in nurses’ job embeddedness

Last, we found that work alienation was an important predictor of job embeddedness and that it could independently explain 10.3% of the variation in job embeddedness after controlling for socio-demographic characteristics. The profiles of work alienation were negatively associated with job embeddedness; nurses in the moderate and high work alienation groups had significantly lower levels of job embeddedness than nurses in the low work alienation group. The lower the degree of work alienation of the nurses, the higher the degree of job embeddedness. The results support our hypothesis and are consistent with previous studies [55, 56]. The possible reason may be that nurses with low work alienation tend to maintain a positive emotional state. Individuals are more inclined to have high levels of autonomy and self-drive to engage in work activities [15], and are more likely to perform their work tasks better and gain a sense of well-being, value, and accomplishment, resulting in a significant increase in job embeddedness.

In contrast, nurses with moderate to high levels of work alienation had lower levels of embeddedness. This may be because work alienation can make nurses think mundanely, believing that work is only a means of obtaining money. By focusing only on the material rewards of work, it is difficult to feel proud and inspired by their caregiving and to approach their work with a dedicated mindset. In addition, higher work alienation often means lower work motivation. Low work motivation can drive individuals to adopt negative work behaviors and attitudes, such as burnout and work distraction, which are more likely to manifest negative exchange relationships and lack of job embeddedness, thus weakening their ties and bonds with the organization [57]. Therefore, managers can guide nurses to use positive psychology to deal with their work, fully mobilize their intrinsic motivation, and maximize well-being and job embeddedness.

In the present study, work alienation profile accounted for 10.3% the variance in job embeddedness, and thus there may be additional predictors of job embeddedness. First, prior research has shown that personality can determine a person’s behavior and thoughts, with agreeableness, openness, and extroversion being significant positive predictors of perceived professional benefits [58]. We hypothesized that there may be a correlation between personality and job embeddedness, but the scientific evidence has yet to be validated. Second, psychological factors may be an important predictor. Several studies have shown that self-efficacy [59], professional self-concept [60], psychological capital [61], emotional intelligence [62], and psychological resilience [63] were significantly related to job embeddedness. There may be additional psychological factors (e.g., silent behavior) that predict job embeddedness, which need to be further tested. Third, research has shown that organizational work factors such as job demands, work-family conflict [64], leader-member exchange [65], organizational climate [39], and job resources [66] affect nurses’ job embeddedness. El-Gazar et al. also found that a person-job match in terms of value, equity, community, and control domains of work life promoted nurses’ job embeddedness [7]. The fourth is the managers’ leadership style and behavior. A study showed that humble leadership could enhance the level of embeddedness [38]. Another study indicated that empowering leadership increased autonomy and responsibility, thus contributing to nurses’ embeddedness in the job [67]. It is not clear how other leadership styles of nursing leaders (e.g., transformational leadership, ethical leadership, shared leadership, inclusive leadership) affect nurses’ job embeddedness. The above factors were not considered here, and further studies are needed to explore more predictors of job embeddedness.

### Limitations and future directions

Several limitations should be considered when interpreting our findings. First, the cross-sectional nature of the research limited our ability to uncover a causal relationship between work alienation and job embeddedness. Further longitudinal studies or interventional studies are needed in the future to verify this association. Second, this study is limited in generalizability; our sample was recruited from three hospitals in Northwest China and is not nationally representative. The participants also contained only clinical registered nurses in China. Due to cultural and institutional differences, the results of this study warrant caution when generalizing to other clinical registered nurses from diverse backgrounds. It will be interesting to examine the impact of cultural differences on the results of this study. Third, the self-reported web-based questionnaires were used in our study, so the results might have been influenced by recall and social desirability biases. Future survey might consider using third-party scorers, e.g., leaders, co-workers, supervisors etc. To improve the representativeness of nurses for subsequent studies, random sampling, and large-scale survey are recommended in future surveys.

Even though existing research on work alienation and job embeddedness has made some progress, there is still work left to do. Work alienation and job embeddedness are complex and dynamic processes involving multidimensional systems of within and outside of work. It makes sense for future research to use longitudinal designs, such as latent transition analysis or growth mixture model, to explore the changes in embeddedness and alienation over time. Quantitative data can also be combined with qualitative data obtained through observations and interviews, contributing to more detailed and realistic results. In addition, other characteristics (e.g., personality traits and organizational work factors) should be included in the study of job embeddedness to gain a more comprehensive picture of the antecedents and outcomes. Future research could also construct targeted interventions to provide ideas for enhancing nurses’ embeddedness, decreasing their turnover intentions, and promoting the development of nursing practice.

### Implications

Despite some limitations, this study has several implications for theory, practice, and nursing education. For theoretical implications, this study extended the existing literature on work alienation and job embeddedness using LPA. Specifically, this study identified the existence of three subgroups of high, medium, and low job alienation, presenting preliminary evidence of the group sensitivity in work alienation. Next, we found a significant relationship between the unique patterns of alienation and embeddedness among nurses. Overall, the findings of this study add novel contributions to reducing nurse turnover and maintaining the vitality of nursing teams.

As for practice contribution, nursing management get to know how the three alienation levels co-occur in their daily clinical work. This might lead to an initial awareness on how they interact with nurses. The results of our study can support the development and implementation of tailored intervention programs for work alienation subpopulations of Chinese nurses. First, for profiles where there is substantial room to diminish in terms of the levels of alienation, for example high alienation group, nursing administrators should identify nurses’ work alienation early. Managers should receive formal training on how to effectively regulate and manage their negative emotions at work, instead of directing them towards abuse of our nurses [23]. Communication could be particularly beneficial, for example to make them understand under-resourcing and underpayment. For nurses with moderate work alienation, nursing manager should pay attention to nurses’ mental health and psychosocial health, and try to meet nurses’ external needs, such as wages, bonuses, work environment, scheduling, or personal development training. It is also highly important to create a harmonious, cordial, fair, and open working atmosphere, which can enhance mutual understanding and support among nurses, satisfy their emotional needs such as a sense of security and sense of belonging, and enhance their level of job embeddedness. Concerning low work alienation profiles, intervention strategies that will sustain these levels can be applied. Powerlessness takes precedence over the other dimensions, i.e., helplessness and meaninglessness, and needs extra attention. Managers should enhance the work autonomy of nurses, develop training programs according to the characteristics of each nurse, and train nurses to become research nurses, specialist nurses, and nursing managers. Adopting innovative strategies can encourage nurses increase their sense of professional value and embeddedness.

In terms for nursing education, nurse educators should understand different development needs based on their demographic characteristics and level of alienation profiles through symposia, interview, and training diaries. In addition, nursing educators need to pay attention to nurses’ continuous professional development and raise awareness of the four career development paths for nurses (clinical, research, teaching, and management). Nurses’ career planning interconnects hospital development goals and nurses’ personal goals, which is conducive to enhancing nurses’ sense of belonging to the organization and embedding them in their work with a higher level of enthusiasm [68].

## Conclusions

Work alienation is a very common phenomenon in the field of nursing. This descriptive cross-sectional online survey found that work alienation had different classification features among Chinese nurses, and different profiles of work alienation had a negative impact on nurses’ job embeddedness. Nursing managers should pay attention to the differences in individual work alienation status, and adopt reasonable management strategies, to improve the level of job embeddedness, ensure the quality of care, reduce nursing turnover, and alleviate nursing shortage.

### Supplementary Information

**Additional file 1.** STROBE Statement—Checklist of items that should be included in reports of cross-sectional studies.

## Data Availability

The datasets analyzed during the current study are available from the corresponding author on reasonable request.

## Abbreviations

AIC: Akaike Information Criterion
aBIC: Sample-size Adjusted Bayesian Information Criterion
BIC: Bayesian Information Criterion
BLRT: Bootstrap Likelihood Ratio Test
COR: Conservation of Resources
IQR: Interquartile range
LMR: Lo–Mendell–Rubin Test
LPA: Latent profile analysis
NNLE: National Nursing Licensure Examination
SD: Standard deviation
SET: Social Exchange Theory
SNK: Student-Newman-Keuls
STROBE: Strengthening the reporting of observational studies in epidemiology
